# Dairy Products with Certification Marks: The Role of Territoriality and Safety Perception on Intention to Buy

**DOI:** 10.3390/foods10102352

**Published:** 2021-10-02

**Authors:** Vincenzo Russo, Margherita Zito, Marco Bilucaglia, Riccardo Circi, Mara Bellati, Laura Emma Milani Marin, Elisabetta Catania, Giuseppe Licitra

**Affiliations:** 1 Department of Business, Law, Economics and Consumer Behaviour “Carlo A. Ricciardi”, IULM University, 20143 Milan, Italy; vincenzo.russo@iulm.it (V.R.); margherita.zito@iulm.it (M.Z.); 2 Behavior and Brain Lab IULM, Center of Research on Neuromarketing, IULM University, 20143 Milan, Italy; marco.bilucaglia@iulm.it (M.B.); mara.bellati@ibba.cnr.it (M.B.); laura.emma.milani@gmail.com (L.E.M.M.); sissicatania1995@gmail.com (E.C.); 3 CNR National Research Council of Italy, 20133 Milan, Italy; 4 Dipartimento Agricoltura Alimentazione e Ambiente (Di3A) Università di Catania, 95123 Catania, Italy; glicitra@unict.it

**Keywords:** dairy products, territoriality, safety perception, intention to buy, multi-group analyses

## Abstract

Over the years, the territorial origins of agri-food products have become a consolidated marketing model which stand as an alternative to mass production. References to territory, whether on packaging or in advertising, have become an increasingly popular way for marketers to differentiate products, by attributing specific characteristics to them, derived from specific cultural identities and traditions. The aim of this study is to capture the possible differences between two groups, Italian and French, in the perception and intention to buy products with certification marks. We tested a multi-group structural equations model, assessing the mediation of the Perceived Product Safety (PPS) between Packaging with reference to Territoriality (PT) and Intention to Buy (IB). Our findings show that in both groups PT has a positive association with IB and PPS and that PPS has a positive association with IB. The difference is the mediation of PPS, present only in the Italian group. This opens important considerations on the role of the perception of safety, particularly in the pandemic period, in the presentation of products, particularly in products with certification marks linked to sustainability and territoriality.

## 1. Introduction

In recent years, there has been a progressive increase in consumer awareness as consumers have become more informed and more demanding with regard to the quality of agri-food products. This has led to the emergence of a growing market for products with a strong territorial identification [1]. The data of the XVIII Ismea-Qualivita Report [2] show that the demand for local or traditional foods is increasing, as they are often perceived to be of higher quality [3], more sustainable, [4] and bearers of a strong cultural identity [5].

Over the years, the emphasis on the territorial origins of agri-food products has become a consolidated marketing model, posing as an alternative to mass production [6,7,8]. References to territory, whether on packaging or in advertising, have become an increasingly popular way for marketers to differentiate products, by attributing specific characteristics to them, derived from specific cultural identities and traditions. Indeed, as stated by Bryła [7], “It is possible to copy all aspects of a food product, but it is impossible to change its history”. Thus, the geographical origin of the product becomes an added value that enables small and medium-sized enterprises to compete with large international companies [9].

References to territoriality can be considered as a driver for the purchase of food products [10,11,12]. The added value of references to territorial origins has led the European Union to adopt a package of legislation (EC Regulations 2081/92 and 2082/92) which provides protection of food names according to their origins: the Protected Designations of Origin (PDO), the Protected Geographical Indications (PGI), and the Traditional Specialities Guaranteed (TSG) [13]. On the consumers’ side, these labels represent a guarantee of quality, since references to territoriality are evocative of concepts that encourage the choice of these products. First, regional products are linked to the concept of tradition, understood as the transmission of knowledge from one generation to the next [14]. Guerrero and colleagues [15] defined a traditional food product as ‘‘a product frequently consumed or associated with specific celebrations and/or seasons, normally transmitted from one generation to another, made accurately in a specific way according to the gastronomic heritage, with little or no processing/manipulation, distinguished and known because of its sensory properties, and associated with a certain local area, region, or country’’. The limited production area and the specificity of the territory contribute to endow the product with special characteristics in the eyes of consumers [9]. Another concept associated with local or traditional foods is that of authenticity [16,17], considered as one of the main drivers in consumers’ attitudes towards brands and products [18,19]. Moreover, another key aspect determining the appeal of local products is their sustainability, that is, the use of production processes that are able to respect the environment and to provide forms of support and jobs for local communities [20,21]. Finally, another function of controlled indications of origin is to reduce the information asymmetry between producers and consumers, so that the latter can always be aware of fundamental aspects regarding the origin and the production of food [22]. For the reasons listed above, brands with certification marks are perceived by the consumer as natural, authentic, safe, and controlled. Van Dijk and colleagues [23] confirmed in their research the fundamental importance of the certification marks as a symbol of protection connected to the territory.

The role of packaging is essential to communicate the product visually and its connection with the territory. Indeed, packaging is the first visual element that puts consumers in contact with the product, a pre-requisite for information processing [23]. It is, therefore, important to investigate the importance of packaging in communicating aspects such as tradition and territoriality [10]. Indeed, packaging could play an important role in the challenge to communicate the abstract concept of “traditional” to new targets, such as young generations.

Until now, research has mostly focused on the perceptual characteristics that packaging must have to convey quality and safety. Specifically, the study by Simmonds et al. [24] suggested that transparent packaging “increased willingness to purchase, expected freshness, and expected quality, as compared to packaging that used food imagery instead. In addition, people expected the products to be tastier, to be more innovative, and were more liked overall in several of the product categories that were assessed.” Chandran et al. [25] found that transparent packaging increased the product trust. However, as specified by Simmonds et al. [24], to have a positive effect on purchase intentions, the product contained in transparent packaging must be visually attractive in order to avoid the opposite effect.

Few empirical studies have instead focused on the role that references to territoriality on packaging have on the willingness to buy and on the psychological mechanisms underlying this choice [10]. Since it is recognized that consumers are willing to pay more for better quality and healthier products [26,27,28,29], in this study we investigate the role played by references to territoriality on packaging of dairy products with a certification mark by hypothesizing that:

**Hypothesis 1a** **(H1a).** Reference to territoriality on packaging has a direct and positive association with intention to buy.

Among the drivers for people to buy territorial products is the sense of safety associated with food, namely the access to healthy food with no risks to human health and no contaminants [30]. According to Espejel and colleagues [31], consumers infer from PDO labels a safety badge, due to the strict controls to which products under the protection are submitted by the regulatory councils. So, we hypothesize:

**Hypothesis 1b** **(H1b).** A positive and direct association of packaging with reference to territoriality with food safety perception.

Moreover, according to Grunert [32], the perception of safety plays a mediating role between the demand and supply of agri-food products. Therefore, according to [33] (but see also [23,31]), we hypothesize:

**Hypothesis 2** **(H2).** A positive and direct association of food safety perception with intention to buy and that:

**Hypothesis 3** **(H3).** Perceived product safety has a mediating role between the sense of territoriality evoked by packaging and the intention to buy.

For our research question we have chosen to focus on the specific category of products from the dairy sector. We chose to focus on dairy products for two reasons. First, dairy products are characterized by a higher contribution to climate change with respect to vegetable foodstuff production, and the relationship between perceived sustainability of dairy products and willingness to pay has already been investigated in the literature [11]. Second, in the European Union, 231 cheeses have a designation of origin [34]. In particular, it is the southern European countries (e.g., Italy, France, Spain) that have the largest number of products that are candidates for registration as PDO or PGI, and these countries are comparable to each other in their familiarity with controlled origin products [14,35]. This makes dairy products suitable for conducting a multi-group survey involving subjects from different European countries, with the aim of testing a first pilot general model or identifying any differences worth further investigation. Herein, we carried out a multi-group study, basing our survey on a sample of Italian subjects and one of French subjects.

**Figure 1:**
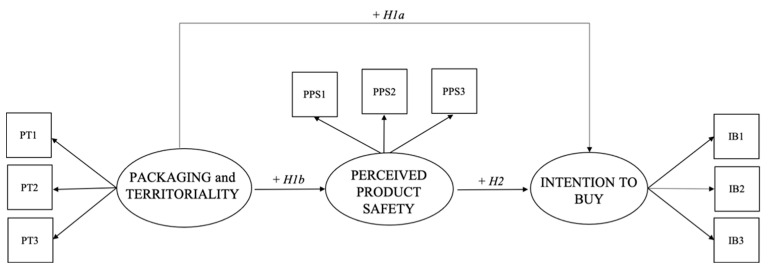
The hypothesized theoretical model.

The following, Figure 1, shows the theoretical model and the expected relationships through the hypotheses.

## 2. Materials and Methods

### 2.1. Participants and Procedure 

The study involved cheese consumers from Italy (IT; N = 400) and from France (FR; N = 200). These two countries were selected on account of the production of dairy products with certification marks. Participants completed a questionnaire placed on an online platform (Google Moduli) to which researchers added a note with instructions to fill in the questionnaire and a note to ensure anonymity. The questionnaire was administered between May and June 2020. Before the questionnaire, participants were asked to answer to some screening questions related to the consumption of cheese (that is, if they bought and ate cheese), the frequency of consumption and purchase, and whether they had food allergies, in particular, cheese allergies. Participants not satisfying the criteria of real consumption of cheese (buying, eating, and the possibility of consuming cheese without allergies) were not considered in the study. Moreover, all participants provided their informed consent in a specific box before filling in the questionnaire. 

The IT sample included 60% females and 40% males, with an average age of 45 years (SD = 10.64). Among them, 11% lived alone, 72.8% were married, and 16.3% lived with their family of origin, and they had an average number of children of 0.780 (SD = 0.932). Their overall cheese consumption (coded as: 2 = rarely, 3 = sometimes, 4 = often) was 3.655 (SD = 0.563). 

The FR sample included 60% females and 40% males, with an average age of 46 years (SD = 12.39). Among them, 19% lived alone, 78% were married, and 4% lived with their family of origin, and they had an average number of children of 0.915 (SD = 1.069). Their overall cheese consumption was 3.710 (SD = 0.536).

The IT and FR samples did not significantly differ in mean age, as shown by the two-sample t-test (χ^2^ (598) = 0.918, *p* = 0.359), nor in number of children (W = 42,020.5, *p* = 0.274) or average cheese consumption (W = 4197.0, *p* = 0.211), as shown by the Mann–Whitney U tests. 

As this study used convenience samples, we compared the demographic data of the two samples. According to ISTAT data, in Italy the updated distribution of females and males in the considered range of age is, respectively, 50.4% and 49.6%, whereas in France it is 51.6% and 48.4%. Even if these data are more gender distributed than the samples of the study (they are more equally distributed than the data of the study, which have a slightly higher percentage of females), they reflect a convergence between the two compared populations, making them comparable (that is, balanced distribution of female and male with slight predominance of females). This balance was also reflected in the samples of the present study, respecting the proportion of the general population and balancing, therefore, the contribution of type/gender in the study as well. 

### 2.2. Measures

On the basis of the literature above mentioned [26,27,28,29,31,32], the study detected three main measures to assess consumers’ attitudes towards the dairy products with certification marks (see the complete list of items in Table 1). 

The first measure is *Packaging and Territoriality* (PT). Starting from the considerations on the role of packaging and the relationship with territoriality [26,27,28,29], we formulated three items as follows: “The packaging must reflect the tradition of production”; “The packaging must recall the territory”; and “The packaging must clarify the place of production”. Participants had to indicate their agreement or disagreement with each item using a 7-point Likert scale ranging from 1 (strongly disagree) to 7 (strongly agree). This measure obtained a very satisfactory reliability, with a Cronbach’s alpha coefficient (α) of 0.82 (IT = 0.86; FR = 0.80).

The second measure is *Perceived Product Safety* (PPS). This measure focuses on the perceptions of consumers of how healthy and nutritious a product is. Three items were, therefore, formulated on the basis of the literature considering the need for safe products, particularly when considering dairy products with certification marks and the considerations of aware consumers [31,32]. The items were as follows: “It is important that the products with the certification mark I choose are healthy”; “It is important that the products with the certification mark I choose are nutritious”; and “It is important that the products with the certification mark I choose are without additives”. Participants had to indicate their agreement or disagreement with each item using a 7-point Likert scale ranging from 1 (strongly disagree) to 7 (strongly agree). This measure obtained a satisfactory reliability, with a Cronbach’s alpha coefficient (α) of 0.72 (IT = 0.70; FR = 0.75).

The third measure is *Intention to Buy* (IB), understood as the positive attitude towards buying dairy products with certification marks. This measure is based on the consideration of the quality that a product should have and the possibility for consumers to place their trust in that product [23,31,32]. Also in this case, three items were formulated: “Thinking about the certification mark, I am willing to pay more”; “Thinking about the certification mark, if it weren’t present in one shop, I would look for it in another”; and “Thinking about the certification mark, I would recommend purchasing it”. Participants had to indicate their agreement or disagreement with each item using a 7-point Likert scale ranging from 1 (strongly disagree) to 7 (strongly agree). This measure obtained a satisfactory reliability, with a Cronbach’s alpha coefficient (α) of 0.78 (IT = 0.80; FR = 0.72).

### 2.3. Data Analyses

Data analyses were performed through SPSS 27 for descriptive statistics, correlations (Pearson’s r), and alpha reliabilities (α) for each scale. These analyses were performed on each group considered in the study. The multi-group structural equations model (SEM) was estimated with MPLUS 8 in order to simultaneously test in both groups, Italian and French, the relationship between the detected variables and the possible presence of a mediation by the perception of product security between the packaging with territoriality links and intentions to buy products with certification marks. It has to be specified that the hypotheses were specified a priori and a partial mediation model was performed [33]. The goodness of fit of the model was evaluated by the chi-square value (χ^2^), the comparative fit index (CFI), the Tucker–Lewis index (TLI), the root mean square error of approximation (RMSEA), and the standardized root mean square residual (SRMR).

To assess possible effects of common method bias, Harman’s single-factor test was performed [36] through a confirmatory factor analysis. Results obtained with MPLUS 8 showed the following fit indices: χ^2^(28) = 709.499, *p* < 0.001, CFI = 0.67, TLI = 0.58, RMSEA = 0.20, SRMR = 0.17. These indices show that the model could not be identified, thus indicating that one single factor did not account for the variance in the data and suggesting that common method bias was unlikely.

## 3. Results

From a psychometric standpoint, all the assessed variables in the study showed satisfactory Cronbach’s alpha values, ranging between 0.70 and 0.90, meeting the criterion of 0.70 [37].

Correlations are shown, for each group, in Table 2 (IT) and in Table 3 (FR), with descriptive statistics of the detected measures. Both samples show high levels of PT and PPS, with means over the central point of the scale (higher levels for the IT sample), whereas the IB variable shows lower levels (with higher levels for the FR sample). As for correlations, the two samples show similar trends in correlation values, in particular for the correlation between PT and PPS (*_r_*_IT_ = 0.63; *_r_*_FR_ = 0.62), with higher values in the FR sample as for the correlation between PT and IB (*_r_*_IT_ = 0.45; *_r_*_FR_ = 0.56) and between PPS and IB (*_r_*_IT_ = 0.42; *_r_*_FR_ = 0.47).

An analysis of variance between the IT and FR samples showed two main significant differences. The IT sample perceived higher levels of PPS (*t*(−2.234) = 410.453 *p* < 0.05), whereas the FR sample showed higher levels in IB (*t*(2.303) = 598 *p* < 0.05).

**Figure 2:**
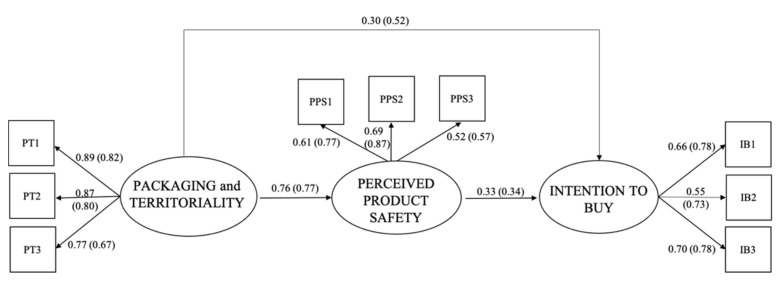
Results of the multi-group structural equations model. Note: Group 1 = IT—outside brackets; Group 2 = (FR)—in brackets.

The estimated multi-group SEM (Figure 2) showed satisfactory fit indices, which confirmed the goodness of the model fit: χ^2^(52) = 132.991 (contribution χ^2^_*IT*_ = 86.119; χ^2^_*FR*_ = 46.872), *p* < 0.00, CFI = 0.96, TLI = 0.95, RMSEA = 0.07 (95% C.I.: 05; 07); SRMR = 0.05. Moreover, the multi-group SEM showed significant and good item loadings (*p* < 0.001) in each group, suggesting a good structure of the latent variables created with these groups of items. In this model, PT showed a direct, positive, and strong association with PPS (*β*_IT_ = 0.76; *β*_FR_ = 0.77) and IB (*β*_IT_ = 0.30; *β*_FR_ = 0.52), particularly in the FR group, confirming H1a and H1b. Moreover, PPS showed a positive and significant association with IB (*β*_IT_ = 0.33; *β*_FR_ = 0.34), in agreement with H2.

Moreover, the model detected the mediating role of PPS between PT and IB. As shown in Table 4, a significant mediation was found only in the IT group, whereas in the FR group this mediation was not significant (*β*_IT_ = 0.25; *β*_FR_ = *n.s*.), partially confirming H3.

## 4. Discussion and Conclusions

In the present study we conducted a multi-group survey with the aim of investigating the relationship between the constructs of PT, PPS, and IB. Based on previous literature, we hypothesized that the perception of rootedness in the territory, evoked by packaging, directly and positively influenced the perception of product safety and the intention to purchase dairy products (H1a and H1b). As underlined, packaging has a key role in communicating the product and in capturing the interest and the attention of consumers [10]. This is linked with the fact that the packaging is a visual stimulus, creating a first contact with the product, a pre-requisite for the processing of information [23]. As for the visual stimuli and the information processing, studies highlight that the visual element is crucial, since the majority of information processed in the brain is mainly visual [38,39]. Moreover, visual elements are considered more significant and reliable, and the use of visual stimuli can help the process of meaning building. From a practical implication standpoint, it would be useful to suggest to producers the use of immediate images and, in this case, the use of clear images and elements linked to territoriality, in order to evoke the interest and traditionality of a specific territory. As highlighted above, these elements can contribute to endow products with special characteristics in the eyes of consumers, such as authenticity [16,17] and both economic and social sustainability [20,21]. In addition, regulations on controlled designations of origin reduce the information gap between producer and consumer [22]. All these elements increase the sense of protection associated with products being rooted in a specific territory. In light of these considerations and of the obtained results, we can argue that the presence of territoriality can enhance the interest of the consumer who evaluates the product as safe, controlled, and traditional, becoming a driver for the decision to buy that product showing a reference to a specific territory [10,11,12]. We also hypothesized that the perception of safety directly and positively influenced purchase intention (H2). By means of a recursive SEM model, we confirmed these research hypotheses both on a sample of Italian subjects and on a sample of French subjects. This would confirm the consumers’ perception of safety as a driver to buy. This is particularly important if territorial products are considered, since safe products are associated with healthy food with no risks to human health and without elements that can contaminate products [30]. In this sense, there emerges the role of communicating the element of safety also through territoriality, so strongly associated with intention to buy, both in the literature and in this study. This also opens an interesting element of deepening the role of culture, considering the mediation results found in this study.

In fact, we also hypothesized that PPS played a mediating role between PT and IB (H3). This research hypothesis was confirmed only in the Italian sample. There are several possible explanations for this result. Firstly, Italy was one of the countries hardest hit by the pandemic, which had a higher mortality rate there than the average for other countries [36]. This situation may have resulted in the Italian sample being more sensitive to the issue of perceived safety, taking into account the period in which the survey was conducted. This would be in line with the result related to the association between PT and IB. In fact, looking at this impact, in the French sample the impact of PT on IB was greater than in the Italian sample, in which the mediation of PPS was present. This would be consistent in explaining the need for safety information in the Italian sample.

Secondly, an explanation for the difference may be found in culturally different associations with the concept of “traditional food”. In fact, Guerrero et al. [35] reported different associations that Italian and French consumers made after hearing the word “traditional”. The French connected it with the words “tasty”, “family”, and “dinner”, while Italians connected it with “home-made”, “natural”, and “old”. The associations made by the French sample refer more to a concept of food quality and moments of conviviality in which food is consumed; the associations made by the Italian sample refer more to production processes and to aspects which the literature mentioned in this paper has associated with the concept of safety. Finally, it should be noted that both samples were shown packaging for Italian products. The issue of ethnocentrism [40] may have influenced the different perception of safety and the role this played in determining the propensity to buy. Future studies could investigate this further by showing both samples regional products typical of both nationalities.

Barcaccia and colleagues [41] reported how the pandemic severely affected the market for typical agri-food products in Italy, generating a surplus of supply and a drastic drop in prices. Our results suggest that a strategy to help the sector cope with the severe economic crisis could be to emphasize references to territoriality, since the perception of territoriality is directly linked to the propensity to buy. Such a strategy could create a virtuous circle with beneficial effects for local food companies and the economy as a whole. Indeed, local/regional tourism uses local food or beverages both to enhance the tourism experience and to support the tradition of local food/beverage production [42].

In conclusion, our results show that the perception of territoriality leads to a greater propensity to buy and a greater sense of perceived safety. In the Italian sample, the safety perception was shown to play a mediating role between the perception of territoriality and the propensity to buy.

A first limitation of this study was the use of only two groups. Future studies should investigate the influence of territoriality and of the perception of safety, in relation to health, on other groups as well. This would allow the determination of differences and fluctuations among different cultures and markets in order to give the right positioning to dairy products with certification marks. Moreover, another limitation of this study was related to the use of a cross-sectional design, as this did not allow us to define causal relationships between variables. Future studies would use neuromarketing techniques to capture the gap between the rational side and the experience and the emotional experience of the subject in real time by having reliable results not mediated by cognitive processes [43]. These techniques in particular would be functional in capturing the role of territoriality in conveying important and useful elements by optimizing the communication and the presentation of the products [10]. This would have a double advantage: producers would have more elements to sell their products and to tell their story and territory (with important consequences for the territory to which they belong [10,42]), and consumers would have more information, easy to find, and a better perception of health and safety. Furthermore, as stated above, other studies aiming at investigating cultural differences in the perception of territoriality on packaging should control for the effect of ethnocentrism by administering products of both nationalities to both samples. 

Another limitation was the use of a convenience sampling method. This procedure allowed us to collect honest and open answers from participants [44] but not to generalize the data. Future studies should provide specific samples both from cultural and from sociodemographic standpoints. However, from a methodological point of view, the sample sizes were adequate to perform a structural equations model, according to the methodological advice suggesting a minimum sample size of 200 [45,46]. 

Moreover, further studies are needed to test the generalizability of our findings to other types of traditional food products. Furthermore, our results suggest the need to further investigate the role played by the perception of safety in determining the propensity to buy typical local products. Future research should also investigate the links between the concept of safety associated with food and the other drivers mentioned above (namely, heritage, authenticity, and sustainability).

## Figures and Tables

**Table 1:** List of measures and items.

Measure	Items
Packaging and Territoriality	1. The packaging must reflect the tradition of production 2. The packaging must recall the territory 3. The packaging must clarify the place of production
Perceived Product Safety	1. It is important that the products with the certification mark I choose are healthy 2. It is important that the products with the certification mark I choose are nutritious 3. It is important that the products with the certification mark I choose are without additives
Intention to Buy	1. Thinking about the certification mark, I am willing to pay more 2. Thinking about the certification mark, if it weren’t present in one shop, I would look for it in another 3. Thinking about the certification mark, I would recommend purchasing it

**Table 2:** Means, Standard Deviations, and Correlations (Pearson’s r) of the IT group.

	M	SD	1	2	3
1. PT	5.90	1.28	(0.86)		
2. PPS	5.63	1.08	0.63 **	(0.70)	
3. IB	3.65	0.97	0.45 **	0.42 **	(0.80)

Note: ** *p* < 0.01. Cronbach’s alphas are on the diagonal (in brackets).

**Table 3:** Means, Standard Deviations, and Correlations (Pearson’s r) of the FR group.

	M	SD	1	2	3
1. PT	5.44	1.31	(0.80)		
2. PPS	5.43	1.05	0.62 **	(0.75)	
3. IB	3.74	0.83	0.56 **	0.47 **	(0.72)

Note: ** *p* < 0.01. Cronbach’s alphas are on the diagonal (in brackets).

**Table 4:** Indirect effects of the estimated multi-group SEM.

Indirect Effects	Standardized Indirect Effects		
	Est.	s.e.	*p*
PT→PPS→IB	0.25 (*n.s*)	0.06 (0.14)	0.00 (0.08)

Note: Group 1 = IT—outside brackets; Group 2 = (FR)—in brackets.

## Data Availability

All data available upon request.
